# AI for social good

**DOI:** 10.1098/rsos.241809

**Published:** 2025-06-11

**Authors:** Philip Treleaven, Daniel Brown

**Affiliations:** ^1^ Department of Computer Science, University College London, London, UK

**Keywords:** GenAI, AI, education, social good

## Abstract

This article describes the Generative AI Education (**GenAIE)** programme: using generative AI (GenAI) to provide personalized education to disadvantaged people, notably probationers and prisoners. For the UK Probation and Prison Service, GenAI (Introducing ChatGPT, 2025, OpenAI; see https://openai.com/blog/chatgpt (accessed January 2025)) is providing education for felons to help stop them reoffending. The UK has over 80 000 prisoners and education is the best deterrent to reoffending, which costs £18bn ($23b) pa (Reoffending Costs, UK Parliament, 2022; see https://questions-statements.parliament.uk/written-questions/detail/2022-0309/137323 (accessed January 2025)). The AI ‘tsunami’ led by GenAI will be hugely disruptive for business and society. However, it also offers pioneering opportunities for social good, notably through personalized education/training for socially excluded and disadvantaged groups (e.g. people on probation, people in prison, refugees, long-term unemployed, long-term sick, low-aspiration young people); thereby transforming their future and addressing major social problems. As a starting point, University College London and MegaNexus are working with educational professionals to produce personalized training content specifically for the Justice sectors, including probation and prisons, described below, which evidences and demonstrates the positive power of GenAI for social benefit. This is part of our *AI for Social Good* programme. As of 31 November 2024, the GenAIE programme had gained over 53 400 users and accumulated over 596 600 hours of Education, Training and Employment learning. We are now working with local councils to support their social services key workers and their clients. To make our paper self-contained but concise, key technical terms are defined as bullet points.

## 1. Introduction

For context, we are now on the threshold of the AI (cf. Fourth Industrial) Revolution. Thirty years ago, we had the IT (cf. Third Industrial) Revolution: where it was essential for everyone to learn IT skills, such as Microsoft Office, to future-proof themselves. The AI revolution will be characterized by specialized AI *assistant* tools controlled by a human user, interacting through natural language processing (text, speech, image, video) [1,2]. The AI assistant will do the preliminary work, such as generating draft content, and answer questions; with the human professionals verifying and completing the material. As an illustration of AI tools supporting professionals, a legal AI assistant (cf. Harvey.AI) might draft a contract, give comments on potential ambiguities and risks; with the expert human lawyer reviewing and correcting the contract. This is referred to as ‘human-in-the-loop’.

Importantly, generative AI (GenAI) can positively impact society in at least five ways:

— **Professional productivity tools**—AI assistants for professionals (e.g. lawyers, accountants, architects, teachers, civil servants and social workers). An example being the legal AI assistant described above.
— **Consumer ‘self-help’ tools**—AI assistants to support consumers in achieving things for themselves. Examples are pension advice, council benefits, advice on litigation or consumer rights.
— **Education tools**—specialized chatbot-driven education and training tools. For example, a GenAI tool trained on a specific textbook with a chatbot user interface, whereby the student can hold a dialogue with the book.
— **Lifestyle enhancement**—a ‘catch-all’ for AI assistants to improve individual’s well-being. Examples include avatar friends for children, digital companions for care home residents, tech advice for the elderly, etc.
— **Social good**—personalized education/training for *socially excluded people* (e.g. prisoners, refugees, long-term unemployed, long-term sick, vulnerable adults, young people in poorly performing schools).

There are many threats ascribed to AI, but the intended contribution of this article is to discuss *AI for Social Good*, introducing underlying AI technologies; discussing role model science, technology, engineering and mathematics (STEM) [3] education platforms supporting teachers and students; and presenting the Generative AI Education (**GenAIE**) platform supporting key workers to help probationers and prisoners. Our mission is twofold:

— **AI-empower key-worker professionals**—through ‘hands-on’ AI assistance using GenAIE and the moderation of client educational content.
— **AI-educate vulnerable users**—with personalized AI-generated educational content to potentially transform their lives.

It is essential when supporting vulnerable people that key-worker professionals are both empowered by AI assistants and have complete control to augment and moderate the content generated (cf. human-in-the-loop).

Vulnerable and disadvantaged people also require personalized support since they typically are poorly educated, socially challenged, and have limited English. Likewise, their educational content needs to be moderated, for example, stopping prisoners seeking GenAI advice on escaping from prison!

## 2. AI for social good

Thirty years ago, society experienced the IT (cf. Third Industrial) Revolution. Now as they say: ‘the AI genie is out of the bottle’. Society is on the threshold of the AI (cf. Fourth Industrial) Revolution with its huge impact on business, employment and students’ education. Textbooks will come with a chatbot tutor. A lawyer will no longer write a contract from scratch; a GenAI tool will produce a draft contract, identifying the key elements and risks for the legal professional to review (cf. human-in-the-loop). Clearly, this constitutes a huge disrupter for the professions and their support staff. Furthermore, and interestingly, aspiring programmers are already using GenAI (plus no-code and low-code tools) and tools such as ‘GitHub Copilot’ for prototype programs. Arguably, everyone will benefit from basic programming skills to read and customize their programs.

As discussed, our focus for the AI revolution is helping key-worker professionals to produce personalized education/training content for their disadvantaged clients.

The core AI education/training ingredients being brought into the mix include:

— **Socially excluded**—focusing on providing AI assistants for key-worker professionals and education/training tools for socially excluded people; the lowest social stratum in a country or community, consisting of the vulnerable, poor, sick and unemployed.
— **AI assistants**—GenAI tutors producing moderated contents for our socially excluded community.
— **Human-in-the-loop**—self-drive AI tools for professionals, a blend of supervised machine learning and active learning where humans are involved in both generating the training material and testing stages of building personalized educational content.
— **Key workers**—professionals in control of the AI assistants, providing moderated educational/training material and monitoring its usage.

Table 1 illustrates the various communities we seek to address. We should also include supporting the elderly, disabled, mentally ill, home carers and single parents.

**Table 1.** AI education/training for social good.

ecosystem	prisons	probation service	refugee counsellors	education	social workers	charities
education/training	career training	career training	social training	inspiring disadvantaged children	care workers	support
key-worker professionals	Ministry of Justice	probation workers	case worker	teachers	social workers	charity workers
socially excluded groups	prisoners	probationers	refugees migrants	low aspiration students	carers in the home	drug addiction alcohol abuse betting addiction

## 3. STEM education platforms

STEM education platforms for supporting teachers and students provide exemplars for what we are seeking to achieve with our GenAI assistants. STEM education, at its core, simply means educating students in four specific disciplines, namely, science, technology, engineering and mathematics (collectively shortened as STEM).

There are over 600 UK organizations involved in STEM education, from professional institutions to learned societies, universities, museums and science discovery centres, specialist education enrichment providers, teacher professional development organizations and subject-specific associations [3]. The STEM education support offered by these organizations covers the full range of primary, secondary and further education, vocational education, apprenticeships and university programmes. The provision ranges from teacher continual professional development (CPD); talks and lectures; classroom-based as well as extra-curricular activities to enhance and enrich the STEM curriculum; specialist and subject-specific educational resources; STEM degree and career pathway mappings and resources and much more.

The main aim of STEM education platforms and networks is to inspire, inform and encourage young people to consider STEM degrees and careers and to help strengthen and diversify the UK STEM workforce. To achieve this, they focus on the following key areas:

— Increased understanding and attitudes towards engineering among young people and their key influencers (e.g. parents, teachers, carers and youth workers).
— Increasing diversity and widening access to under-served communities and under-represented groups.
— Increasing and offering high-quality support and further development for teachers of STEM subjects, through innovative teaching and employer engagement methods.
— Embedding STEM throughout primary and secondary education with a focus on early interventions.
— Supporting teaching and learning in further education and promoting technical and vocational routes into STEM.
— Development of specialist resources focusing on advice and guidance on STEM career and degree pathways across different sectors, including applying to university, work experience, research and industry placements.

STEM students are likely to be high achievers. In contrast, using GenAI tools for low-aspiration and disadvantaged (often invisible) people takes education to a whole new level.

## 4. AI algorithms

**Figure 1.**
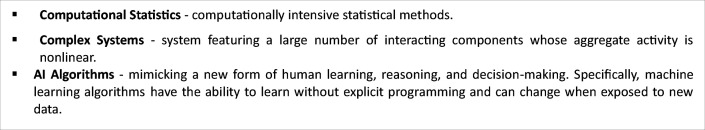
Algorithm taxonomy [4].

To position GenAI (e.g. ChatGPT), algorithms cover three broad domains: computational statistics (e.g. Monte Carlo methods), complex systems (e.g. agent-based systems) and artificial intelligence, specifically machine learning (e.g. artificial neural networks). See figure 1.

At a simple level, AI machine learning covers:

— **Traditional AI**—ML models to identify patterns within a training dataset and make predictions.
— **Generative AI**—GenAI is used to describe any type of AI used to dynamically create new ‘human-like’ texts, images, speech, video, programs or synthetic data.

And for the future:

— **Algorithmic general intelligence (AGI**)—solving problems as well as humans; faced with an unfamiliar task, the AGI system could find a solution.
— **Algorithmic superintelligence (ASI**)—solving all problems better than people across a comprehensive range of categories and fields of endeavour.

### 4.1. Generative AI

ChatGPT, Llama, Mistral, Gemini, DeepSeek and over 30 GenAI models have a proven capability of holding human-level conversations but are also subject to generating inaccurate, unethical and misinformation. Fascinating is *AI hallucination*: a confident response that is biased, too specialized, even totally wrong [5]. These AI hallucinations can be caused by various factors, but the most common is when the model has limited training data and has not been programmed to say: ‘I don't know that answer’. Instead of saying that, it will make something up that seems like it could in fact be the answer. This illustrates the huge importance of the key-worker professional ‘human-in-the-loop’ driving the AI algorithm.

OpenAI’s ChatGPT is acknowledged as a leap in innovation (cf. iPhone) with GPT-4 trained on 170 trillion parameters. Plug-ins are now available using Microsoft 365 Copilot and GitHub, etc., with numerous companies launching GenAI ad-ins to their products, such as Bloomberg GPT [6]. And on the horizon is algorithmic superintelligence (ASI) far surpassing most gifted human minds.

In general, GenAI is a broad label for any type of AI used to dynamically create new ‘human-like’ content [7]. GenAI has massive implications for business, education, employment, but also law enforcement and crime. As commented by the UK Sunday Times newspaper [8] ‘the ability to entwine a chatty, randy, bot with the image or video of a [real] person made to order to meet a user’s preferences is [already] here’.

Key GenAI terms are:

— **Generative pre-trained transformers (GPT**) − a family of AI models that uses the Transformer architecture and is a key advancement in AI powering GenAI applications such as ChatGPT [9].
— **Large language models (LLM**) − LLMs are a subset of AI trained on a vast quantity of (online) data to produce human-like responses to dialogue or other natural language inputs [10].

## 5. Review of generative AI tools

Here, we briefly review GenAI tools to illustrate innovation. There are literally thousands of GenAI tools entering the marketplace [11]. As discussed, they can positively impact society in a number of ways:

— **Professional productivity tools**—AI assistants for professionals (e.g. lawyers, accountants, architects, creatives, human resources and civil servants).
— **Consumer ‘self-help’ tools**—AI assistants to support consumers in achieving things for themselves. An example is personalized investment, tax returns or pension advice.
— **Education tools**—specialized AI-driven education and training tools, using interactive GenAI.

We highlight some professional and consumer applications and AI tools as exemplars.

### 5.1. Professional GPT applications

In the business area, GenAI is producing a new generation of AI professional productivity tools. Examples include:

— **GitHub Copilot**—a cloud-based AI tool developed by GitHub and OpenAI to assist programmers, accelerating the speed of software development.
— **Harvey.AI**—assists with legal contract analysis, due diligence, litigation and regulatory compliance and can help generate insights, recommendations and predictions.
— **Synthesia**—AI revolutionizing content creation by enabling the generation of lifelike videos using text inputs. A University College London (UCL) ‘unicorn’.
— **Skillate**—streamlines the recruiting process by providing enhanced applicant experiences, intelligent recruiting services backed by AI, and people analytics.

### 5.2. Consumer GPT applications

As expected, there are a growing number of consumer AI tools especially in the creative industries, such as art, music, video and even beauty.

— **Dall-E**—an AI system that can create realistic images and art from a description in natural language.
— **Hemingway**—highlights aspects of ‘poor’ style including overlong sentences, passive voice, and excessive adverb use.
— **Voicify**—an AI-based music creation platform that allows users to generate custom songs with the voices of their favourite artists.
— **Midjourney**—an advanced AI tool renowned for its ability to generate high-resolution images from image or text prompts.

### 5.3. Generative AI models

GenAI or LLM models such as ChatGPT, Llama, Mistral, Gemini and DeepSeek:

— **ChatGPT−4o**—OpenAI’s chatbot that uses natural language processing to create human-like conversational dialogue enabling users to refine and steer a conversation.
— **DeepSeek-R1**—the Chinese AI model that offers comparable performance to the world’s best chatbots at seemingly a fraction of their development cost.

The plethora of AI tools and apps is multiplying and already supporting increasing volumes of professionals and consumers alike. However, while they have on the one hand been accompanied by many prophesies of machines overrunning human beings, on the other hand they have not been at all focused on supporting those who could benefit the most: the socially excluded and disadvantaged groups at the fringes of society who do not have the resources to seek them out.

## 6. Generative AI training and risks

Given the socially excluded community we are addressing, AI model training and risks are paramount. GenAI tools are notorious for hallucinations, deepfakes, cybercrime*,* inaccurate information, and misuse.

Hence the AI tools we have developed support key-worker professionals to produce, monitor and regulate the personalized educational/training content, rather than giving their clients free rein. As mentioned, if prisoners are given uncontrolled access to a GenAI chatbot, they can ask ‘what skills do I need to become a chef?’ or equally ‘what do I need to escape from prison?’. In computing, this is referred to as a *walled garden,* providing an environment that moderates the user’s access to network-based content and services. In effect, the walled garden directs the user’s navigation within particular areas to enable access to a selection of material and prevent access to other material.

### 6.1. AI training data

Training data refer to the massive datasets used to teach AI models to detect patterns, make predictions and generate new information. The performance and propensity for incorrect responses, misinformation and *hallucinations* of an AI system hinge critically on the quality and diversity of its training data [5]. If data used lacks diversity or contains biases, the AI can develop blind spots and make unreliable judgements, especially in unfamiliar cases. For example, an AI system trained primarily on images of young, light-skinned individuals may have limited ability to recognize darker-skinned or older faces. In practice, limited data availability, inconsistent labelling, and lack of diverse perspectives can lead to gaps in training data. Continually assessing and strengthening AI training data are crucial to developing systems that produce fair, accurate and trusted results for all. Careful curation must remain a collaborative effort between data scientists, experts and communities affected by AI systems. This is being addressed by retrieval-augmented generation (RAG), a technique for enhancing the accuracy and reliability of GenAI models with facts fetched from external ‘trusted’ sources [12].

### 6.2. AI-generated information

To continue, GenAI is notorious for generating inaccurate information. Here, what is referred to as prompt engineering is the art of crafting prompts that effectively instruct GPT tools to generate the desired output [13,14]. Information challenges include:

— **Opaque information**—hard to understand; not clear or lucid; and obscure generated information.
— **Inaccurate information**—data that are inaccurate, incomplete, or inconsistent, but not deliberately created.
— **Misinformation**—false or inaccurate information, especially that which is deliberately intended to deceive, including false information, which is intended to mislead, especially propaganda issued by a government organization to a rival power or the media.
— **Inappropriate information**—inappropriate content consists of information or images that should not be generated or provided that might lead to unlawful or dangerous behaviour. For example, a prisoner asking for advice on escaping, or a user asking how to make an illegal drug.
— **AI hallucination**—generation of outputs that may sound plausible but are either factually incorrect, or unrelated to the given context; with the algorithms generating additional nonsensical content when challenged by the user [5].

### 6.3. Professional in the loop

The above is the overwhelming reason for the ‘professional in the loop’, to produce educational/training content and ensure that the chatbot-generated information is moderated. Without proper safeguards, such technology could enable, for example, misinformation and ‘deepfakes’ to deceive audiences.

In summary, AI *hallucinations* [5] pose a significant challenge for our domain when an AI model generates a confident yet inaccurate response. The causes of AI hallucinations are multifaceted. Limited or biased data used to train the AI can skew its knowledge and judgements. Lack of data on certain populations or topics can lead to overconfidence in generating erroneous information about those areas.

## 7. GenAIE GPT educational platform

Our GenAIE platform was developed, firstly, to provide public sector key workers with AI assistants; and secondly, to provide eLearning content across the (public sector) educational and vocational spectrum. GenAIE builds upon the MegaNexus Community Campus software used in the UK Probation Service and Prisons.

The Community Campus educational platform provides a secure means of access to selected learning materials at different levels and in multiple languages that align to the needs of each individual learner. This in turn requires GenAI content that is appropriate to the requirements of each individual in terms of vocational area, skills, learning abilities, job aspirations and language. GenAIE supports key-worker professionals to build courses, lessons and resources using GenAI. This accelerates development and supports moderated subject matter delivery. In addition, all content materials can be provided in multiple (foreign) languages important, especially, for users with limited or no English.

The requirement was for a system to support key-worker professionals in automatically building lesson content in different formats and media, and at different levels in different languages, personalized to individuals’ learning abilities and tailored to the key stage level of learning needed. The AI automatically provides lesson plans, resource hyperlinks and extra-curricular activities such as flashcards, quizzes and wordsearches, with the ability for human teachers to edit, amend and insert additional information to the lesson content.

Content also needs to be published in a variety of formats. For example, with Moodle, a course between 2 and 10 modules provides teachers with a backbone of subject knowledge, alleviating lesson preparation time to allow focus on targeted individuals while bringing creativity and life-support to the subject. In addition, AI systems need to provide consistent assessment points to enhance understanding of students’ progress, giving better guidance towards the grades needed to pass and to excel.

GenAIE is a fully automated content generation platform for professionals and their clients. It supports key workers to build, within minutes, courses, lessons and resources inclusive of modules and lessons, extra-curricular resources and audio assistance using GenAI.

This accelerates course material development, supporting moderated subject matter delivery potentially across the educational and vocational spectrum, to enable teachers to best focus on communication with and support for students.

GenAIE can operate either independently or with an existing learning management system (LMS) platform to provide personalized education with learning pathways for each user and automated content provision configured for the needs of each individual. As mentioned, this content is personalized for each individual in terms of vocational area, skills, learning abilities, job aspirations and language with all content materials available in multiple languages, especially significant for disadvantaged users with limited English.

The GenAIE platform utilizes a microservices architecture and GenAI to automate content creation; it is cloud based for scalability. This architecture deploys the application as a collection of services, providing the framework to develop, deploy and customize services independently.

GenAIE leverages a set of different fine-tuned transformer-based (GPT) natural language models. Natural language generation workflows chain together prompt engineering, LLMs and helper agents into pipelines. These are coordinated by different agents or *genies* (curriculum, student, teacher and moderator) with different motivations performing different tasks. They ensure that the content produced is appropriate for a varied set of stakeholders with different needs and wants.

**Figure 2.**
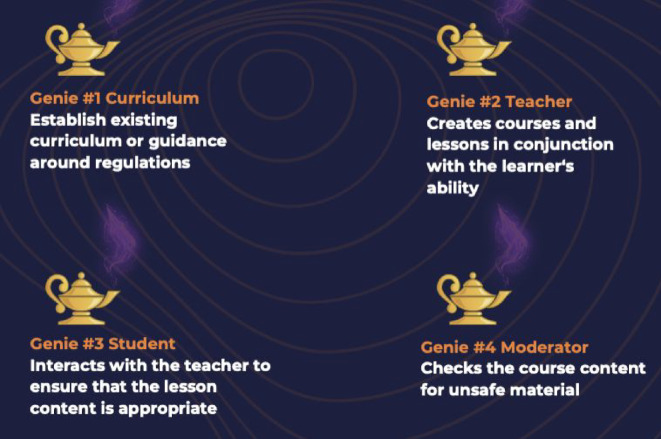
GenAIE course flow based on ‘Genies’.

Figure 2 summarizes course generation flow, built around a collection of ‘genies’, each with a specific function:

— **Curriculum genie**—ensures compliance against known curriculums, legislation and regulations.
— **Teacher genie**—generates lesson plans, learning material, activities, signposted resources.
— **Student genie**—ensures language and content are appropriate for the level expected.
— **Moderator genie**—guardrails around profanity, language and inappropriate materials.

During the production of content, each ‘genie’ operates with the others to ensure that moderated course content aligns to a set curriculum, covers appropriate signposted resources, and accords with students’ expected levels, (vocational) interests and capabilities. To support a key worker in producing educational course material, GenAIE utilizes a multi-step collaboration approach:

— **Curriculum bot**—performs focused LLM retrieval to source related curricula.
— **Teacher genie**—reviews and customizes the curriculum.
— **Outline agent**—uses prompt templating to generate a course outline based on a requested number of lessons.
— **Teacher genie**—refines the outline.
— **Lesson agent**—leverages fine-tuned Text to Text Transfer Transformer (T5) to generate individual lessons.
— **Teacher genie**—edits lessons prior to finalization.
— **Resource agent**—uses AI visual generation tools and LLMs to generate supplemental assets including audio, images and quizzes and multiple languages.
— **Export**—the curriculum packages are exported to an appropriate format (e.g. SCORM) for LMS integration (e.g. pptx for editing, or PDF for print).

### 7.1. Knowledge transfer

In summary, our ‘AI for Social Good’ mission is to empower key-worker professionals and their clients through knowledge transfer using a four-stage process:

— **GenAIE deployment**—UCL and MegaNexus work with educational professionals to produce personalized training content for a specific sector, such as people in prisons and people on probation (PoPs) described below, to demonstrate the constructive power of GenAI.
— **Key worker AI training**—a training and awareness course for key-worker professionals on AI and emerging technologies and their impact on social services. As part of the training, professionals prepare sample educational material using GenAIE.
— **Key worker empowerment**—professionals independently use GenAIE to produce educational material and also monitor its deployment and usage.
— **Client education**—interactive GenAI tutors producing moderated contents for our socially excluded users.

## 8. Case study: GenAIE for people in the probation service

As a case study we look specifically at education for PoPs, and the preparation of personalized educational material by probation specialists. As you will understand are unable to provide detailed statistics due to the sensitivity of the subject matter.

As presented in the abstract, reoffending in the UK has a £18bn ($23bn) pa cost [2] (UK Parliament, 2022) with one of the greatest recidivism influences being lack of education and training. A major challenge facing key-worker professionals supporting learning for probationers is targeting relevant course materials for each individual on their personal pathway and at the right learning level, in an appropriate format with courses that meet their interests and (vocational) needs. The volume of permutations required to supply these materials has historically been very challenging for the Probation Service with the cost of bespoke configuration of content a major obstacle.

Hence the UK Probation Service required a large-scale provision of educational materials for PoPs to support them on a variety of vocational pathways. The key requirement is to provide moderated content generation that keeps expert human educators in control of the course generation process but saves time in developing appropriate effective content at different levels, in different languages calibrated to the capabilities of different learners. The aim is to allow educators to focus on curriculum and learning pathway design and to gauge the enhancements and improvements needed for content and curricula without having to work on the more onerous and time-consuming course materials implementations that are less effective uses of their human skills.

In summary, the GenAIE platform is used by key workers to provide a variety of tailored educational/training materials calibrated to individual learners. Course materials are produced through GenAI using a system developed in a partnership between a team at UCL and MegaNexus to support development of online learning materials.

**Figure 3.**
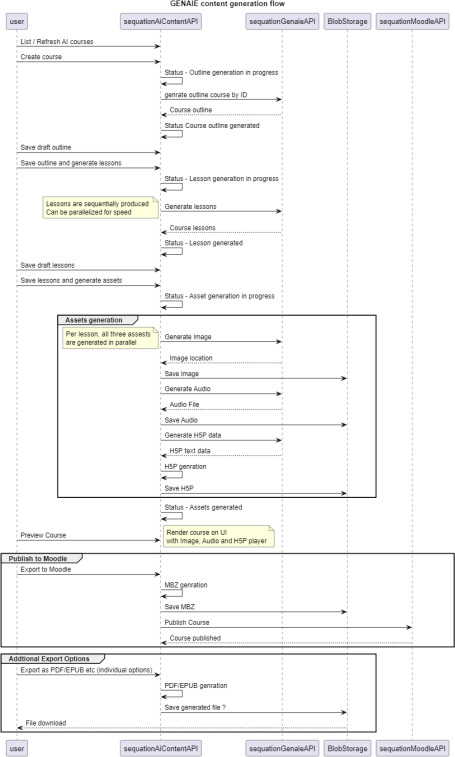
GenAIE course generation flow for probationers.

Figure 3 illustrates GenAIE course development. For the Ministry of Justice, a range of courses were developed to support learners on probation and in prisons using GenAI. These incorporated the adversarial genie approach highlighted above to ensure that content is made appropriate for the audience, targets relevant education levels, satisfying curricula requirements where needed and allows humans in the loop to provide governance and encouragement. All materials produced are subject to human educator approval before being passed into appropriate formats on the Community Campus. This inclusion of humans in the loop is a key form of governance and an effective use of people resources providing a critical guardrail and which is far more cost effective and efficient than generating course materials from scratch.

Firstly, course subject matter is selected by the Head of Education, Training & Employment Improvement (HETEI) at the UK HM Prison & Probation Service (HMPPS) National Community Payback Team. GenAIE then develops the course materials in text format. These are reviewed by the HETEI and added to or amended where appropriate. Following this, GenAIE develops the full course content materials including audio and video materials in multiple languages. These are then automatically exported in a structured format to the secure Community Campus LMS.

For the Probation Service, GenAIE provided a series of nine complete introductory courses for PoPs through an Internet-accessible platform portal available online through the Community Campus. The primary objective of these courses is to improve the employability of individuals engaged in unpaid work.

As discussed, GenAIE created customized courses for Community Campus supporting PoPs. These courses have adopted an animated style and feature a diverse range of characters representing varied demographics, reflecting the diversity of today’s society. Moreover, all courses are available in 10 languages, aligning with HMPPS’s cohort. Community Campus is accessible across England and Wales, supporting over 53 400 users with over 596 600 h of education, training and employment learning.

We would like to have included a detailed scientific analysis of the impact. However, the Ministry of Justice is highly sensitive to external contact with their probationers and prisoners. Hence, we have merely included anecdotal qualitative feedback received from both key workers and PoPs, as highlighted by these examples:

‘Community Campus has been a real benefit to Probation, it is something that’s been required for a long time, it has taken us away from the fluffy courses that we didn’t have any control of’.

Community Payback Operational Manager.

‘I have always wanted to do a plumbing course, and this has given me the start’.

Person on Probation regarding Introduction to Plumbing course.

In summary, GenAIE has significantly reduced the time and effort required for resource creation, leading to significant cost savings while effectively addressing the personalized needs of UK HMPPS. Following the success of GenAIE in supporting PoPs, HMPPS adopted the GenAIE platform to support people in prison.

**Figure 4.**
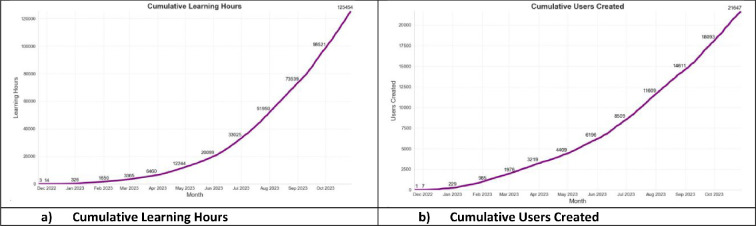
GenAIE educational impact on probationers. (a) Cumulative learning hours. (b) Cumulative users created.

The graphs in figure 4 show the level of engagement received since the launch in December 2022. UK HMPPS has seen exponentially increasing results in terms of active users and has delivered over 596 600 h of learning.

## 9. Conclusions

In keeping with the UK Government’s priority for AI, *AI for Social Good* is a significant social development in personalized education/training for disadvantaged groups of people. Thereby potentially transforming their futures and addressing major social problems. As introduced, this includes PoPs and in prison, but also major cohorts of vulnerable people with limited opportunities. It offers a notable pathway for AI technology. It is already operating across the Probation Service and Prisons, plus schools, Further Education colleges and refugee centres. As highlighted, the cost to society of reoffending in the UK alone is estimated at £18bn ($23bn) per annum. GenAIE offers the opportunity to positively impact the reoffending cycle at low cost and further to prevent the origins of offending behaviour in the first instance.

For the future, we seek additional partnerships with professionals in other areas including local authority Social Services staff, and Jobcentre staff seeking to tackle long-term unemployment. We also wish to explore lifestyle enhancement for the aged, disabled and care community.

Our focus on the socially excluded in society is proving remarkably rewarding.

## Data Availability

This article has no additional data.
